# Refeeding Syndrome May Increase the Risk of Anemia of Prematurity: Is Early Enteral Nutrition the Solution?

**DOI:** 10.3390/nu18091380

**Published:** 2026-04-28

**Authors:** Maria Di Chiara, Caterina Spiriti, Gaia Loffredo, Fabiana Russo, Daniela Regoli, Cecilia Betto, Francesco Crispino, Gianluca Terrin

**Affiliations:** 1 Department of Maternal and Child Health, Policlinico Umberto I, Sapienza University, 00161 Rome, Italygianluca.terrin@uniroma1.it (G.T.); 2 UOC Pediatria e Neonatologia, P.O.A. Cardarelli, ASReM, 86100 Campobasso, Italy

**Keywords:** anemia, refeeding syndrome, hypophosphatemia, parenteral nutrition, enteral nutrition, preterm infants

## Abstract

Background/Objectives: Preterm infants are particularly vulnerable to nutritional deficiencies and electrolyte imbalances during the early stages of extrauterine life. To ensure optimal metabolic support, they often require the early initiation of “aggressive” parenteral nutrition (PN), which is a known risk factor for Refeeding Syndrome (RS), a potentially serious metabolic condition characterized by fluid and electrolyte disturbances, the most significant of which is hypophosphatemia. Hypophosphatemia can impair the metabolism, survival, and function of red blood cells, leading to a reduction in key intracellular metabolites and the development of a metabolic block that alters their quality and decreases their stability. It is therefore hypothesized that RS may contribute to the development of anemia of prematurity (AOP). At the same time, early enteral nutrition (EN) may promote metabolic adaptation and reduce exposure to the complications of prolonged parenteral support, potentially protecting against AOP. The primary aim of this study was to determine whether preterm infants who develop RS are at increased risk of AOP. A secondary aim was to evaluate whether early EN may act as a protective factor against the development of AOP. Methods: This retrospective observational study was conducted on infants with a gestational age ≤ 34 weeks and/or birth weight ≤ 1500 g, admitted to the Neonatal Intensive Care Unit of Policlinico Umberto I—Sapienza University of Rome, between January 2015 and November 2022. Infants diagnosed with AOP were classified as cases, while those without AOP served as the control group. Results: A total of 412 preterm infants were enrolled (110 cases, 302 controls). Refeeding Syndrome was significantly more frequent in infants with AOP (30.9% vs. 11.6%, *p* < 0.001). In the logistic regression model adjusted for gestational age, RS was independently associated with AOP (OR = 2.81; 95% CI: 1.55–5.10; *p* < 0.001), along with gestational age ≤ 34 weeks (OR = 7.10; 95% CI: 2.13–24.0; *p* = 0.001). Early enteral nutrition during the first week of life was associated with a significantly lower risk of AOP (OR = 0.12; 95% CI: 0.029–0.52; *p* = 0.005). The association between RS and AOP was confirmed in the model adjusted for birth weight (OR = 2.06; 95% CI: 1.16–3.79; *p* = 0.021). Infants with AOP showed significantly higher parenteral nutrition intake, delayed initiation of enteral feeding, and later achievement of full enteral nutrition compared with controls (all *p* < 0.001). Conclusions: RS is significantly associated with AOP in preterm infants, likely through pathophysiological mechanisms related to hypophosphatemia. Importantly, early EN may be a protective factor against AOP, suggesting that timely initiation and advancement in enteral feeding may counteract the metabolic derangements associated with intensive parenteral support. These findings support a nutritional approach that prioritizes early and progressive enteral nutrition as a strategy to reduce the risk of both RS and AOP. Further prospective studies are needed to confirm these associations and to define optimal EN protocols for this population.

## 1. Introduction

Preterm infants are particularly exposed to a high risk of morbidity and mortality, especially in the early stages of extrauterine life, when they are extremely vulnerable to nutritional deficiencies and electrolyte imbalances [1,2]. To counteract this phenomenon, evidence recommends an early and enhanced nutritional strategy from the very first hours of life [3,4]. Recent evidence, however, suggests that this type of nutritional strategy is associated with an increased risk of developing Refeeding Syndrome (RS), a metabolic condition characterized by electrolyte disturbances, the hallmark of which is hypophosphatemia [5,6,7]. The latter represents one of the most common metabolic complications in this fragile population and is characterized by sudden fluid and electrolyte imbalances, the most typical of which is hypophosphatemia [8,9].

The intestinal immaturity of preterm infants further complicates nutritional management, as the evolving gut requires careful modulation of nutrient delivery to support both systemic growth and intestinal barrier function [10,11,12]. At the same time, nutritional management in preterm infants is not limited to parenteral support. In very preterm newborns, early enteral feeding has been associated with better tolerance of PN and fewer early metabolic complications, supporting the view that earlier advancement in enteral nutrition (EN) may reduce exposure to the metabolic disturbances related to intensive parenteral support [2]. Robust evidence supports the early introduction of trophic enteral feeds in preterm infants. Even at minimal volumes, early EN promotes maturation of the intestinal mucosa and of gastrointestinal motility, activates the enteroinsular axis, and favors the development of a protective microbiota, thus contributing to a lower risk of necrotizing enterocolitis and late-onset sepsis [2].

Whether this nutritional strategy may also protect against AOP, however, remains unclear. The literature highlights that hypophosphatemia can impair erythrocyte metabolism by reducing essential metabolites and increasing osmotic fragility and oxidative stress in red blood cells [13,14,15]. In preterm infants, early enhanced amino acid administration has been shown to drive a rapid intracellular shift in phosphate, precipitating early hypophosphatemia in the first days of life [16].

Indeed, phosphate is a key substrate for glycolytic metabolism and ATP synthesis, which are the primary energy sources for mature red blood cells. It is also essential for the production of 2,3-diphosphoglycerate (2,3-DPG), a critical regulator of hemoglobin–oxygen affinity, and for the maintenance of cell membrane integrity through phospholipid turnover. Furthermore, phosphate depletion compromises antioxidant defenses, exposing erythrocytes to oxidative damage and premature destruction. Considering that RS is common in preterm infants and that phosphate is thus involved in numerous cellular processes essential for the production, metabolism, and survival of red blood cells, we hypothesized that RS may represent a potential risk factor for the onset of anemia of prematurity (AOP).

On these grounds, the hypophosphatemia observed during RS could negatively affect red blood cell production, function, and survival in preterm infants, potentially contributing to the development of anemia of prematurity.

The primary aim of our study was to investigate a possible relationship between the development of Refeeding Syndrome and the onset of anemia of prematurity (AOP). A secondary aim was to evaluate whether early EN may serve as a protective factor against the development of AOP.

## 2. Materials and Methods

### 2.1. Study Design

The study was designed as a case–control study, dividing the population into two groups: the cases, represented by newborns diagnosed with anemia of prematurity (AOP), and a control group consisting of newborns who did not develop the condition. In this study, anemia of prematurity (AOP) was defined as a normocytic, normochromic, hyporegenerative anemia typical of preterm infants. In accordance with the literature, the following threshold values were used as diagnostic criteria to identify significant anemia: hemoglobin < 8 g/dL or hematocrit < 25%, generally reached between the fourth and sixth week of life in newborns with a gestational age of less than 34 weeks [17,18].

### 2.2. Study Population

All preterm newborns with a gestational age (GA) ≤ 34 weeks and/or birth weight < 1500 g, consecutively admitted to the Neonatology, Pathology, and Neonatal Intensive Care Unit of Policlinico Umberto I, Sapienza University of Rome, were considered eligible. All newborns presenting one or more of the following conditions were excluded from the study: severe clinical conditions at birth; major congenital malformations; inborn errors of metabolism; congenital infections; intraventricular hemorrhage grade ≥ 3; death or transfer to another hospital within the first 72 h of life.

### 2.3. Nutritional Protocol

All newborns included in the study received parenteral nutrition (PN) within 24 h of admission to the Neonatal Intensive Care Unit (NICU), following the placement of a central venous line (CVC) or a peripherally inserted central catheter (PICC). PN was administered with the goal of providing an adequate supply of fluids, electrolytes, and nutrients until the achievement of full enteral nutrition (FEN), defined as 120 kcal/kg/day. The PN prescription was made daily by the responsible neonatologists, tailored to each infant based on clinical conditions and laboratory results, using dedicated software.

The macronutrient and micronutrient content of PN was calculated according to the local nutritional protocol. Calcium infusion (10% calcium gluconate) was initiated on the first day of life at a dose of 40 mg/kg/day and was progressively increased to 80 mg/kg/day by the eighth day. Similarly, phosphorus infusion (sodium glycerophosphate) began on day 1 at 25 mg/kg/day and was gradually increased to 75 mg/kg/day by the eighth day, maintaining a phosphorus-to-calcium ratio of 1:1. Enteral nutrition (EN) was started with minimal enteral feeding (10–20 mL/kg/day divided into 4–8 feedings), as soon as the infant’s general clinical condition allowed. Beginning at 48–96 h, in the absence of feeding intolerance during the previous 24 h and in clinically stable newborns, the local protocol recommended a daily increase in EN of 15–30 mL/kg/day, adjusted according to birth weight. Early EN was operationally defined as the achievement of an enteral intake exceeding 70% of the total daily nutritional intake (enteral + parenteral) during the first week of life. Total enteral volume over postnatal days 1–7 was divided by total fluid intake over the same period; infants reaching the 70% threshold were classified as receiving early EN.

### 2.4. Data Collection

Neonatal data referring to the period between admission to the Neonatal Intensive Care Unit and discharge, transfer, or death were collected and stored in a dedicated database. Prenatal, perinatal, and postnatal data were examined and recorded. Daily data on macronutrient and micronutrient intake through parenteral nutrition were collected using a specific data sheet by physicians who were blinded to the study objectives. Anthropometric parameters (both standardized and non-standardized) were collected and reviewed prospectively. Percentiles and Z-scores for birth weight, length, and head circumference were calculated according to the growth charts of the Italian Neonatal Study (INeS).

The diagnosis of Refeeding Syndrome (RS) was made in the presence of simultaneous hypophosphatemia (P < 1.6 mmol/L) and hypercalcemia (ionized Ca > 1.3 mmol/L), occurring within the first 2 weeks of life [16,19].

### 2.5. Ethics

The study was conducted in accordance with the Declaration of Helsinki of the World Medical Association for medical research involving human subjects and was approved by the Ethics Committee of Policlinico Umberto I, Sapienza University of Rome. Written informed consent was obtained from both parents prior to inclusion in the study.

### 2.6. Statistics

Statistical analysis was performed using the Statistical Package for Social Sciences (SPSS) software for Microsoft Windows (SPSS Inc., Chicago, IL, USA), version 27.0. The normality of distribution was assessed using the Shapiro–Wilk test. Continuous variables with normal distribution were summarized as mean and standard deviation and compared using Student’s *t*-test; non-normally distributed variables were expressed as median and interquartile range and compared using the Mann–Whitney U test. Categorical variables were compared using the chi-square test. The significance level was set at *p* < 0.05 (two-tailed test). A binary logistic regression analysis was performed to evaluate the independent association between Refeeding Syndrome and Anemia of Prematurity. Covariates entered into the multivariable models were selected on the basis of statistical significance at univariate analysis (*p* < 0.05) and/or documented biological relevance as risk factors for AOP. The following variables were included: Refeeding Syndrome, placental abruption, umbilical cord pH < 7.2, male sex, and early enteral nutrition (enteral volume > 70% of total intake during the first week of life). Because gestational age and birth weight are highly collinear, two separate models were built: Model 1 was adjusted for gestational age (≤34 weeks) and Model 2 for birth weight (≤1250 g). Collinearity between covariates was checked using the variance inflation factor (VIF), with all values <2. A statistician blinded to the study objectives received the database, in which each patient was identified by an anonymous code. A post hoc power analysis was conducted to evaluate the statistical adequacy of the sample size. For the primary comparison of Refeeding Syndrome prevalence between cases and controls (30.9% vs. 11.6%), with an effect size (Cohen’s h) of 0.48, the achieved power was 99.2% (α = 0.05, two-tailed).

## 3. Results

A total of 412 newborns were enrolled in the present study, of whom 110 developed AOP (case group) and 302 did not develop the condition (control group), all with a gestational age ≤ 34 weeks and/or birth weight < 1500 g.

Table 1 reports the baseline clinical characteristics of cases and controls. A higher incidence of placental abruption was observed in the case group (12.7% vs. 6.3%, *p* = 0.033). Statistically significant findings included: acidosis at birth (23.6% vs. 13.6%, *p* = 0.018), significantly lower gestational age in the case group (27.5 vs. 30.3, *p* < 0.001).

**Figure 1:**
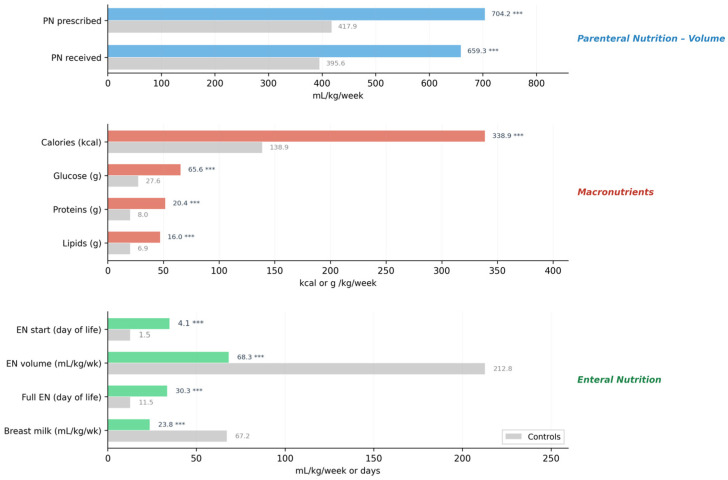
Horizontal bar chart comparing nutritional parameters between infants with Anemia of Prematurity (AOP, Cases, *n* = 110) and controls (*n* = 302) during the first week of life. Variables are grouped by category: parenteral nutrition volume (blue), macronutrients (red), and enteral nutrition (green). Controls are shown in grey. Values represent medians. All comparisons were statistically significant (*** *p* < 0.001, Mann–Whitney U test).

Figure 1 summarizes the main nutritional parameters during the first week of life, highlighting that infants with AOP received significantly higher volumes of parenteral nutrition, greater macronutrient intake, and had a delayed start and lower volume of enteral nutrition compared with controls (all *p* < 0.001). Phosphorus intake from parenteral nutrition was also significantly higher in the case group (392.1 vs. 199.9 mg/kg/week, *p* < 0.001), reflecting the greater overall PN volume and longer duration of parenteral support in these infants.

RS occurred in 34 of 110 Cases (30.9%) and in 35 of 302 Controls (11.6%), a statistically significant difference (*p* < 0.001).

**Figure 2:**
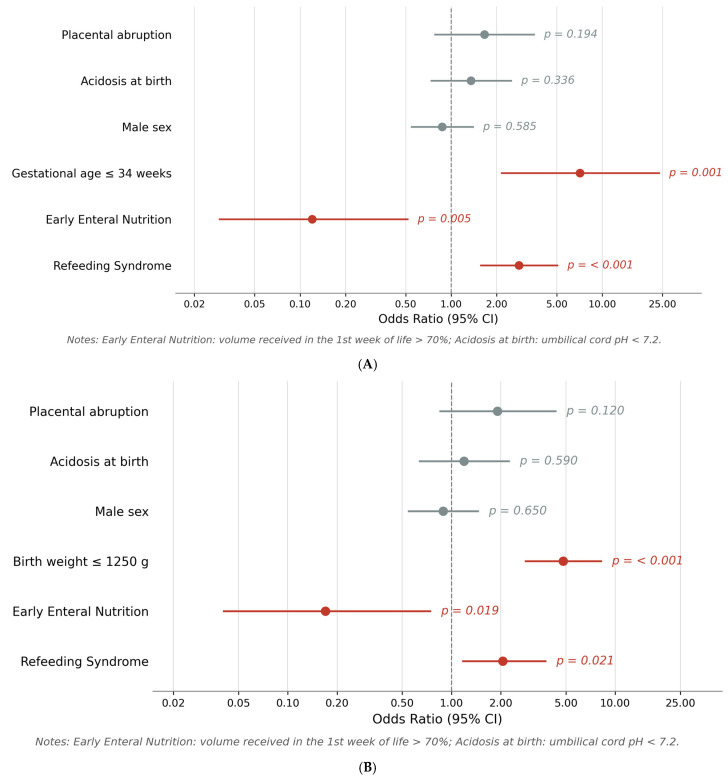
(**A**). Forest plot showing the results of the binary logistic regression analysis adjusted for gestational age. Odds ratios (OR) with 95% confidence intervals (CI) are displayed on a logarithmic scale. Significant predictors (*p* < 0.05) are shown in red; non-significant variables are shown in grey. The dashed vertical line indicates OR = 1 (null effect). *Notes: Early Enteral Nutrition: volume received in the 1st week of life > 70%; Acidosis at birth: umbilical cord pH < 7.2.* (**B**). Forest plot showing the results of the binary logistic regression analysis adjusted for birth weight. Odds ratios (OR) with 95% confidence intervals (CI) are displayed on a logarithmic scale. Significant predictors (*p* < 0.05) are shown in red; non-significant variables are shown in grey. The dashed vertical line indicates OR = 1 (null effect). *Notes: Early Enteral Nutrition: volume received in the 1st week of life > 70%; Acidosis at birth: umbilical cord pH < 7.2*.

The binary logistic regression analyses are reported in Figure 2A,B. In both models, Refeeding Syndrome remained independently associated with AOP after adjustment for the main confounding factors. Receiving enteral nutrition > 70% of total intake during the first week of life emerged as a significant protective factor against AOP. Placental abruption, acidosis at birth, and male sex were not independently associated with AOP in either model.

## 4. Discussion

RS, alongside gestational age and birth weight, is an independent risk factor associated with AOP, but early enteral nutrition acts as a protective factor against the development of AOP. Taken together, these findings suggest that the way nutritional support is delivered in the first days of life may influence not only metabolic adaptation, but also subsequent hematologic outcomes in preterm infants. Experimental and clinical studies show that hypophosphatemia, an essential feature of RS, can impair the production, maturation, and survival of red blood cells. However, this evidence primarily derives from studies that evaluated the role of hypophosphatemia on erythropoiesis in isolation, without contextualizing it within the broader pathophysiological framework of RS in preterm infants. To the best of our knowledge, the present study is the first to directly investigate the association between RS and AOP in the neonatal population. While previous research has explored the consequences of RS in preterm infants and separate studies have characterized the risk factors for AOP, no prior work has bridged these two domains [20].

As expected in a preterm population, the Case group showed a lower gestational age than controls. This difference is consistent with the pathophysiological rationale of the study, since the most immature infants are structurally more exposed to prolonged parenteral support, which itself predisposes to Refeeding Syndrome. Gestational age, therefore, acts simultaneously as a non-modifiable determinant of AOP and as an upstream driver of PN exposure and represents the primary confounder of the RS–AOP relationship. For this reason, our analytical strategy was built around two parallel multivariable models that adjusted for the two most informative proxies of prematurity severity. The association between RS and AOP remained independently significant after adjustment for gestational age and was confirmed in the model adjusted for birth weight, supporting the hypothesis that RS contributes to the development of AOP beyond the effect of prematurity itself.

The etiology of Anemia of Prematurity is inherently multifactorial. A blunted erythropoietin response to postnatal anemia, the shorter lifespan of the preterm erythrocyte, rapid postnatal growth with consequent dilution of red cell mass, and the physiological delay in the hepatic-to-renal switch of erythropoiesis together create an intrinsic vulnerability to anemia in this population [21]. These constitutional mechanisms are amplified by modifiable factors such as limited nutrient stores and inadequate intake of iron, folate, vitamin B12, vitamin E and copper, cumulative phlebotomy losses, and the pro-inflammatory milieu of sepsis, necrotizing enterocolitis and bronchopulmonary dysplasia, all of which suppress erythropoiesis. Our findings add a further dimension to this framework, identifying Refeeding Syndrome as a previously underrecognized metabolic contributor to AOP.

Although infants with AOP received higher absolute phosphorus intake, this reflects their greater overall PN volume and longer duration of parenteral support. The development of hypophosphatemia despite higher phosphorus delivery is consistent with the pathophysiology of RS, in which the anabolic demand driven by high protein and glucose intake exceeds the capacity of exogenous phosphate supplementation to maintain intracellular phosphate homeostasis.

An equally relevant finding of this study is the protective role of early EN against the development of AOP. In both logistic regression models, a higher volume of enteral nutrition received during the first week of life was significantly associated with a reduced risk of AOP. This observation is biologically plausible: early enteral feeding promotes intestinal maturation, enhances the incretin response, and has been shown to improve metabolic tolerance to parenteral nutrition, reducing complications such as hyperglycemia, hypertriglyceridemia, and metabolic acidosis [2]. Furthermore, the earlier achievement of full enteral nutrition allows for a shorter duration of parenteral support, thereby limiting the exposure to the metabolic conditions that predispose to RS and its hematological consequences. These findings suggest that the promotion of early and progressive enteral feeding may represent a key modifiable factor in the prevention of AOP in preterm infants. Moreover, by potentially reducing the incidence of AOP and shortening the duration of NICU stay, early enteral nutrition strategies may also mitigate the well-documented psychological burden on families of preterm infants requiring prolonged hospitalization [22].

This interpretation is consistent with our findings showing that infants who developed AOP received higher volumes of PN, greater macronutrient intake, delayed initiation of enteral feeding, and lower enteral volumes during the first week of life. Thus, the protective association of EN should not be viewed simply as the opposite of RS, but rather as a distinct and clinically modifiable nutritional factor that may shape metabolic adaptation in preterm infants.

In the murine model described by Park et al., mice fed a phosphorus-deficient diet initially developed erythrocytosis; however, this was accompanied by a reduction in early erythroid burst-forming units (BFU-E), suggesting an intrinsic alteration in erythroid progenitor differentiation mechanisms [23]. This condition was associated with reduced erythropoietin (EPO) production and increased expression of heme oxygenase (Hmox-1) in the spleen, indicative of increased erythrocyte clearance [23]. Although the methodology adopted by Park et al.—based on a genetically controlled animal model—differs from our clinical-retrospective approach, the results suggest that phosphorus is a key modulator of red blood cell quality and stability.

The absence of an adequate erythropoietic response in the early stages of life in preterm infants may partly be explained by a well-known aggravating factor for anemia of prematurity [24].

Added to this is the physiological immaturity of erythropoietin (EPO) production: in preterm infants, EPO synthesis is still predominantly hepatic, as the transition toward renal production has not yet been completed. This condition results in a hyporegenerative and delayed erythropoietic response, which further contributes to the development of anemia during the first days and weeks of life. In humans, historical studies such as those conducted by Travis et al. and Lichtman et al. showed that hypophosphatemia, in the context of parenteral nutrition, can cause profound alterations in erythrocyte metabolism. In particular, a marked reduction in glycolytic metabolites, ATP, and 2,3-diphosphoglycerate (2,3-DPG) was documented, leading to impaired red blood cell function and increased hemoglobin affinity for oxygen, resulting in inefficient oxygen release to tissues. It should be noted, however, that these studies were conducted in adult populations and do not directly address the neonatal context, let alone that of preterm infants [24,25]. This neurological immaturity is part of a broader vulnerability that predisposes preterm infants to a range of neurological sequelae, including headache disorders at a developmental age, further underscoring the clinical complexity of this population [26].

Studies conducted in bovine animal models have also strengthened the pathophysiological plausibility of a link between hypophosphatemia and erythrocyte dysfunction. In particular, Zhang et al. showed that phosphorus deficiency leads to a reduction in the number of red blood cells and in mean hemoglobin content, accompanied by an increase in oxidative stress [27]. According to our current knowledge, however, no studies are presently available that have directly evaluated the association between RS and AOP in the neonatal population.

Small-for-gestational-age (SGA) and intrauterine growth-restricted (IUGR) infants deserve specific consideration in this context, since chronic intrauterine undernutrition depletes intracellular micronutrient stores and favors an adaptive hyperinsulinaemic state that amplifies the electrolyte shift occurring at the reintroduction of parenteral amino acids and glucose, thereby predisposing these infants to Refeeding Syndrome. In our cohort, however, the prevalence of SGA did not differ between Cases and Controls, indicating that the association between Refeeding Syndrome and Anemia of Prematurity described here is not primarily driven by growth-restricted infants. These newborns nevertheless remain a high-risk population, warranting tailored electrolyte monitoring and a more cautious advancement in parenteral macronutrients during the first week of life.

Beyond the NICU stay, preterm infants and, in particular, those who have experienced early nutritional and metabolic derangements such as RS and AOP remain at increased risk of pediatric readmission after discharge, which further underlines the importance of structured post-discharge follow-up programs aimed at early detection of hematological, nutritional, and growth-related issues and at supporting parental caregiving [28].

### 4.1. Biological Plausibility

A pioneering study on adult patients receiving phosphate-free parenteral nutrition demonstrated that hypophosphatemia can cause profound alterations in red blood cell glycolytic metabolism [24]. In particular, the reduction in plasma inorganic phosphate was associated with a significant decrease in key glycolytic intermediates (glucose-6-phosphate, fructose-6-phosphate, 3-phosphoglycerate, 2-phosphoglycerate, phosphoenolpyruvate), accompanied by marked depletion of 2,3-diphosphoglycerate (2,3-DPG) and adenosine triphosphate (ATP), both essential for erythrocyte function and survival. At the same time, there was an accumulation of triose phosphates (dihydroxyacetone phosphate and glyceraldehyde-3-phosphate), indicating a metabolic block upstream of the reaction catalyzed by glyceraldehyde-3-phosphate dehydrogenase (GAPDH), a phosphate-dependent enzyme.

These observations were later confirmed and expanded by more recent studies, which showed that phosphate deficiency leads to a series of functional and structural alterations in red blood cells. Specifically, the following have been documented: a significant reduction in intracellular ATP and 2,3-DPG levels, resulting in impaired erythrocyte energy metabolism; membrane composition alterations, particularly a reduction in phospholipids, increasing osmotic fragility and the risk of hemolysis; and activation of oxidative stress with reduced glutathione peroxidase activity and increased malondialdehyde (MDA) levels, a marker of oxidative damage to red blood cells, contributing to direct erythrocyte injury [17,27,29].

Another key element supporting the biological plausibility of the association between RS and AOP is fibroblast growth factor 23 (FGF23), a phosphaturic hormone produced mainly by osteocytes and, to a lesser extent, by bone marrow cells. Production of FGF23 increases in response to hypophosphatemia, iron deficiency, inflammatory states, and various other pathological conditions [30]. The literature has documented that FGF23 not only inhibits the synthesis of erythropoietin (EPO) but also promotes apoptosis of erythroid cells and interferes with cell cycle progression of erythroid progenitors, specifically, it reduces the S phase and increases the G2/M phase, resulting in a slowdown of erythroid proliferation and a marked decrease in red blood cell production [31,32,33].

### 4.2. Study Limitations

Limitations of the study should be interpreted. First, the retrospective nature entails constraints related to the availability, completeness, and quality of the clinical and laboratory data collected. Second, since the study was conducted in a single center, the generalizability of the findings to other healthcare settings or neonatal populations may be limited. Third, although the sample size is adequate for the analyses performed, it is not large enough to allow evaluations of specific subgroups or less frequent associations. Fourth, no biochemical data were available on FGF23, hepcidin, 2,3-diphosphoglycerate (2,3-DPG), markers of hemolysis, erythropoietin (EPO), inflammatory cytokines, or markers of oxidative stress, which could have strengthened the pathophysiological interpretation of the findings. Finally, despite the multivariate analysis including the main known factors, the influence of unmeasured variables on the observed association between RS and AOP cannot be excluded. In particular, delayed enteral feeding may partly reflect greater clinical instability, which itself could contribute to worse hematologic outcomes. A further consideration relates to the broader evidence base: the comparability of our findings with previous reports on refeeding syndrome is inherently limited by the marked heterogeneity of clinical settings and target populations characterising existing studies, most of which were conducted in adult or mixed paediatric cohorts with only limited neonatal-specific evidence [33]. Prospective multicenter studies are therefore needed to confirm whether RS plays a causal role in the development of AOP and to clarify whether optimizing early enteral nutrition can reduce this risk.

### 4.3. Clinical Implications

Our findings translate into several concrete recommendations for neonatal clinical practice. First, RS should be recognized as a modifiable risk factor for AOP, not merely as an acute electrolyte disturbance. Second, systematic monitoring of serum phosphate and ionized calcium during the first two postnatal weeks should be implemented in all preterm infants receiving aggressive parenteral nutrition, with particular attention to those with the lowest gestational age or birth weight and to SGA/IUGR infants. Third, the nutritional paradigm in the NICU may benefit from a shift from “enhanced PN/cautious EN” to “gentle PN/aggressive EN”, prioritizing the early introduction and stepwise advancement in enteral feeds whenever clinically tolerated [34]. Fourth, PN composition should be individualized, with specific attention to the amino acid–to–phosphate ratio during the first week of life in order to limit the anabolic demand that drives hypophosphatemia. Finally, infants who develop RS should undergo more intensive hematological surveillance, given their higher risk of subsequently developing AOP.

### 4.4. Public Health and Preventive Implications

From a public health standpoint, our findings reinforce the role of standardized NICU nutritional protocols as a preventive tool. The systematic implementation of early and progressive enteral nutrition, paired with structured biochemical surveillance of phosphate and calcium during the first two weeks of life, could reduce the population-level incidence of both RS and AOP. Such strategies require multidisciplinary teams (neonatologists, pharmacists, clinical nutritionists, nurses, and dietitians) and continuous educational programs to ensure adherence and homogeneity across centers. Indeed, the effectiveness of such educational programs is ultimately shaped by the knowledge, attitudes, and behaviours of the healthcare workforce, a principle consistently documented in other paediatric preventive contexts, and warrants systematic assessment to inform tailored training intervention [35]. At a system level, preventing AOP may translate into fewer red blood cell transfusions, shorter duration of parenteral support and central venous access, reduced length of hospital stays, fewer readmissions after discharge, and a lower psychological burden on families. Taken together, these findings support the integration of a “gentle PN/aggressive EN” paradigm into neonatal care pathways as a simple, low-cost, and potentially high-impact preventive strategy for one of the most common complications of prematurity.

## 5. Conclusions

The findings of this study indicate that RS is significantly associated with the development of AOP in preterm infants. This association suggests that “aggressive” PN may contribute to the development of RS and, consequently, of AOP. Conversely, early EN emerged as a significant protective factor against AOP, reinforcing the concept that the timely initiation and advancement in enteral feeding may counteract the metabolic derangements triggered by intensive parenteral support. These findings carry important clinical implications: in the nutritional management of preterm infants, particular attention should be devoted not only to optimizing PN but especially to promoting early and progressive enteral nutrition as a strategy to reduce the risk of both RS and AOP. Overall, these findings may support a shift in neonatal nutritional strategy: from “aggressive” parenteral nutrition to aggressive EN and gentle parenteral support.

To the best of our knowledge, this is the first study to explore the association between RS and AOP in preterm infants, suggesting a novel pathophysiological link that warrants further investigation. However, to confirm the causal relationship between RS and AOP and to clarify its clinical implications, further prospective, multicenter, and controlled studies are essential.

## Figures and Tables

**Table 1:** Baseline clinical characteristics of neonates enrolled in the study.

	Cases (*n* = 110)	Controls (*n* = 302)	*p* Value
Prenatal characteristics			
Maternal Age ≥ 35 years, *n* (%)	42 (40.4)	132 (48.9)	0.140
Placental abruption, *n* (%)	14 (12.7)	19 (6.3)	0.033 *
Doppler flowmetry abnormalities, *n* (%)	16 (14.5)	52 (17.2)	0.518
Blood pressure disorders, *n* (%)	26 (23.6)	64 (21.2)	0.595
Thyroid disorders, *n* (%)	13 (11.8)	39 (12.9)	0.767
Gestational diabetes, *n* (%)	8 (7.3)	37 (12.3)	0.152
IUGR, *n* (%)	20 (18.2)	44 (14.6)	0.371
Postnatal characteristics			
Twin pregnancy, *n* (%)	29 (26.9)	96 (32.9)	0.173
Cesarean section, *n* (%)	91 (82.7)	256 (87.7)	0.055
Umbilical cord pH < 7.2, *n* (%)	25 (23.6)	38 (13.6)	0.018 *
Male sex, *n* (%)	56 (50.9)	164 (54.7)	0.499
Gestational age, weeks	27.5 (27.0–27.9)	30.3 (30.0–30.6)	<0.001 *
SGA, *n* (%)	22 (20.0)	56 (18.5)	0.738
Birth weight, z-score	−0.38 (−0.6 to −0.17)	−0.38 (−0.52 to −0.25)	0.662
ELBW, *n* (%)	63 (57.3)	38 (12.6)	<0.001 *
Length of hospital stay, days	84.2 (75.3–93.1)	49 (46.2–51.8)	<0.001 *

SGA: small for gestational age; ELBW: extremely low birth weight; IUGR: intrauterine growth restriction. Continuous variables expressed as median (IQR). * *p* < 0.05.

## Data Availability

The raw data supporting the conclusions of this article will be made available by the authors, without undue reservation.
